# Oxygen-releasing biomaterials for osteoarthritis: advances in managing the hypoxic joint microenvironment

**DOI:** 10.3389/fcell.2025.1704327

**Published:** 2025-11-06

**Authors:** Ye Wang, Zan Chen, Wenjie Huang, Mengkun Liu, Renliang Zhao, Xiangtian Deng

**Affiliations:** 1 Trauma Medical Center, Department of Orthopedics Surgery, West China Hospital, Sichuan University, Chengdu, China; 2 Orthopedics Research Institute, Department of Orthopedics, West China Hospital, Sichuan University, Chengdu, China; 3 Department of Orthopedics, Affiliated Hospital of North Sichuan Medical College, Nanchong, China

**Keywords:** oxygen therapy, oxygen-releasing biomaterials, osteoarthritis, bone tissue engineering, hypoxic microenvironment

## Abstract

Osteoarthritis (OA) is a degenerative joint disease characterized by cartilage degeneration and osteophyte formation, with no fundamentally effective therapies currently available. Existing treatments are mainly symptomatic (e.g., drug injections and joint replacement) and cannot reverse the pathological progression, resulting in limited efficacy. A hypoxic microenvironment is a significant barrier to OA treatment: increased inflammatory cells in the synovium lead to higher oxygen consumption, causing cartilage hypoxia that exacerbates inflammation via hypoxia-inducible factors and accelerates cartilage damage. In recent years, research on oxygen-generating biomaterials targeting joint hypoxia has become a hot topic. Such materials continuously release oxygen through mechanisms like peroxide decomposition, enzyme-catalyzed reactions, or photosynthetic microbes, thereby increasing local oxygen partial pressure, relieving tissue hypoxia, and suppressing oxidative stress, which is expected to promote cartilage regeneration. This review systematically explores the hypoxia-induced pathogenic mechanisms of OA, innovatively categorizes and describes the fabrication strategies of oxygen-releasing biomaterials developed in recent years, analyzes their potential molecular mechanisms in OA therapy, and highlights current limitations in oxygen-release controllability and biosafety, as well as future research directions.

## 1 Introduction

**FIGURE 1:**
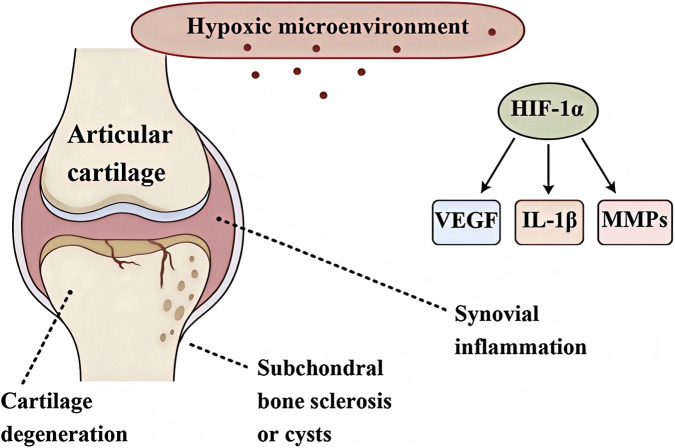
Schematic diagram of the key pathological processes induced by hypoxic microenvironment in osteoarthritis degeneration.

Osteoarthritis (OA) is a common degenerative joint disease whose progression is accompanied by changes in the local pathological microenvironment of the joint, such as abnormal biomechanical stress, accumulation of inflammatory mediators, elevated oxidative stress, and hypoxic conditions (Tang et al., 2025). The hypoxic microenvironment within the joint cavity is considered a crucial pathogenic factor, particularly in certain forms of OA (Figure 1) (Zhang et al., 2025). Normal cartilage tissue is avascular and operates under low oxygen tension, but in OA this balance is disrupted, and dysregulated oxygen supply to chondrocytes has profound effects on joint tissues. Given the beneficial role of oxygen in bone and soft tissue repair, various oxygen therapies have been applied clinically, including (HBOT), local oxygen-enriched devices, near-infrared phototherapy, and wearable photobiomodulation devices such as low-intensity lasers (Feng et al., 2025; Zou et al., 2023). These interventions have been shown to improve joint function to varying degrees by increasing local oxygen supply, but their limited tissue penetration, short duration of effect, and reliance on exogenous oxygen supply greatly restrict their widespread application and long-term efficacy.

With the rapid development of tissue engineering, research on “oxygen-generating biomaterials” that provide self-oxygen supply and modulate the microenvironment in OA treatment has flourished in recent years (Lang et al., 2025). These biomaterials have been proposed to help overcome these issues, especially in advanced or end-stage OA where hypoxia and tissue degeneration are pronounced. By providing sustained, localized oxygen delivery, these biomaterials alleviate chronic intra-articular hypoxia and improve cellular viability and function. This sustained oxygenation can modulate hypoxia-related signaling pathways (e.g., aberrant HIF-1α activity) that otherwise drive cartilage degradation. Moreover, continuous oxygen release supports chondrogenesis and integration of repair tissue with subchondral bone, leading to enhanced osteochondral regeneration even in severe OA cases (Zhang H et al., 2023). This review focuses on exploring the latest advances in oxygen-generating materials, emphasizing their design, functional characteristics, and biological activity. Additionally, the article delves into the critical role of hypoxia in OA pathogenesis, highlighting the key pathological changes it induces. Finally, by systematically comparing the advantages and limitations of different types of oxygen-generating materials, this review aims to point out new directions for the treatment of this challenging clinical problem in OA.

## 2 Hypoxia induced pathological changes in joints

### 2.1 Cartilage degeneration and destruction

Hypoxic conditions exacerbate OA cartilage degeneration and destruction through multiple molecular mechanisms. Under mild hypoxia, the HIF-1α pathway is activated to maintain chondrocyte survival and extracellular matrix (ECM) homeostasis, playing a protective role, however, under sustained severe hypoxia, HIF-1α exhibits a “double-edged sword” effect. Overactivation of HIF-1α induces expression of cartilage matrix-degrading enzymes (such as MMP-13 and ADAMTS-5), accelerating ECM breakdown (Figure 1) (Phillips, 2022).

Meanwhile, hypoxia causes abnormal accumulation of reactive oxygen species (ROS) within chondrocytes and activates the NF-κB signaling pathway, upregulating pro-inflammatory mediators and additional matrix metalloproteinases. This amplifies the local inflammatory response and induces chondrocyte apoptosis. Hypoxia also inhibits the PI3K/AKT/mTOR survival signaling pathway, weakening chondrocyte autophagy and proliferation, such impaired signaling can lead to autophagy dysfunction and hindered cell renewal, reducing the cells’ capacity to cope with stress (Wang et al., 2016).

Furthermore, chronic hypoxia causes mitochondrial dysfunction: it blocks oxidative phosphorylation in chondrocytes, resulting in decreased ATP production and disturbed energy metabolism. Excess ROS production together with energy deficiency induces cellular stress damage. This metabolic imbalance and ROS burden also diminish the ability of chondrocytes to synthesize key ECM components like collagen II and proteoglycans (Zhao Z et al., 2024). Collectively, these hypoxia-mediated molecular events interact and ultimately promote the degeneration and destruction of articular cartilage, accelerating the progression of OA.

### 2.2 Synovial inflammation

The synovial tissue in OA is often under low oxygen, and hypoxia has been proven to be a critical driver of chronic synovial inflammation. At the molecular level, hypoxia can induce synovial macrophages to polarize to the pro-inflammatory M1 phenotype: HIF-1α is stabilized under hypoxic conditions and, together with activation of pathways such as STAT3 and Notch1, drives macrophages to express an M1 phenotype and secrete high levels of pro-inflammatory cytokines (e.g., TNF-α, IL-1β), exacerbating synovial inflammation (Hua and Dias, 2016). Simultaneously, hypoxia causes a large accumulation of intracellular ROS, and excessive ROS can trigger NLRP3 inflammasome activation. This promotes the maturation and release of inflammatory cytokines (especially IL-1β and IL-18) and induces pyroptotic death of synovial cells, thereby establishing a sustained chronic inflammatory state.Additionally, hypoxia directly acts on synovial fibroblasts (FLS), causing their activation and the production of numerous pro-inflammatory and tissue-destructive factors. These include cytokines such as TNF-α, IL-6, and IL-8, and matrix metalloproteinases like MMP-1, MMP-3, and MMP-13. This leads to elevated levels of inflammatory mediators and cartilage matrix-degrading enzymes in the synovial microenvironment, accelerating the degeneration of cartilage and subchondral bone (Kim et al., 2019).

On the other hand, hypoxia also weakens local immune regulation within the synovium. HIF-1α-mediated signaling can inhibit transcription factors such as FoxP3, impeding the differentiation and function of regulatory T cells (Treg) and reducing the production of anti-inflammatory cytokines (such as IL-10). This results in weaker protective immune responses and a shift toward a pro-inflammatory imbalance between Th17 and Treg cells. In summary, hypoxia induces a positive feedback loop of pro-inflammatory factors and immune dysregulation in synovial tissue, driving and sustaining chronic synovitis in OA and promoting disease progression.

### 2.3 Subchondral bone sclerosis and cystic changes

Recent studies have confirmed that hypoxia promotes pathological changes in the (Zhang S et al., 2023). Hypoxia continuously stimulates HIF-1α and its downstream vascular endothelial growth factor (VEGF) expression, driving HIF-1α/VEGF axis-mediated abnormal angiogenesis. This causes abundant new blood vessels (including CD31, EMCN and H-type vessels) to invade the normally avascular subchondral bone region. The formation of these abnormal vascular networks disrupts the normal osteochondral interface barrier, leading to disturbed local oxygen tension and inducing vascular calcification and osteoid matrix deposition. Excessive angiogenesis is often accompanied by an osteogenic response, producing excessive bone formation and ultimately leading to abnormal thickening (sclerosis) of the subchondral bone plate.

Notably, HIF-1α expression is significantly higher in sclerotic subchondral bone compared to relatively healthy areas, further supporting the close association between hypoxic signaling and subchondral bone sclerosis. Simultaneously, hypoxia influences the differentiation fate of bone marrow mesenchymal stem cells (BMSCs), promoting their shift toward the osteogenic lineage rather than the chondrogenic lineage. For example, hypoxic culture enhances BMSC mineralization and increases the expression of osteogenic markers (such as bone sialoprotein and osteocalcin), which may correspondingly weaken their potential for chondrogenic differentiation (Wang H et al., 2025).

Furthermore, under hypoxic and inflammatory conditions, osteoclasts in the subchondral bone become over-activated: early-stage bone resorption is enhanced and trabecular bone mass is transiently reduced, exacerbating an imbalance in bone remodeling. As the disease progresses, osteoclast activity gradually declines, bone resorption is reduced, and osteogenesis dominates, leading to excessive trabecular thickening and architectural disruption. Localized abnormal mechanical stress further results in microfractures and bone marrow edema, forming subchondral bone cystic lesions. These molecular mechanisms illustrate the key role of hypoxia in the development and progression of subchondral bone sclerosis and cystic changes in OA.

## 3 Classification and research progress of oxygen-releasing materials

**FIGURE 2:**
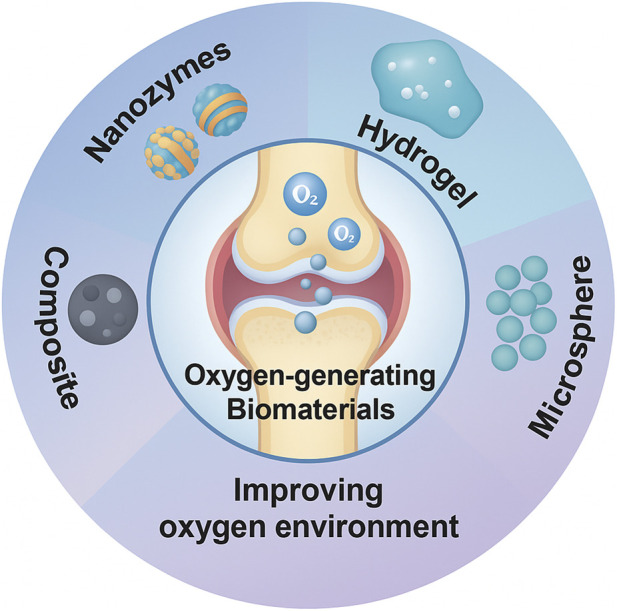
Schematic diagram of the main types of oxygen producing biomaterials in recent years.

Based on the pathological features of joint hypoxia in OA and the joint’s unique anatomy, various “oxygen-generating materials” have been developed for OA repair. The core idea is to release oxygen within the joint cavity to improve local hypoxia while also scavenging excess ROS to protect chondrocytes. These materials mainly include: nanozymes (artificial enzyme-mimetic nanomaterials) (Yu et al., 2024), hydrogels (polymer gels loaded with oxygen sources or carriers) (Wang J et al., 2025), microspheres/particles (slow-release inorganic peroxide particles) (Miao et al., 2024), and composite systems (integrating oxygen-generating components with antioxidative/anti-inflammatory components or scaffolds) (Bordon et al., 2023) (Figure 2).

### 3.1 Nanozyme-based oxygen-releasing materials

Nanozymes are nanomaterials with enzyme-like catalytic activity that can decompose ROS or H_2_O_2_ in the joint to generate O_2_, mimicking natural oxidoreductases (primarily superoxide dismutase [SOD] and catalase [CAT]). Common nanozymes include transition metal oxides (e.g., MnO_2_, CeO_2_), noble metal nanoparticles (e.g., Pt, Pd), and their composites. They efficiently catalyze the conversion of H_2_O_2_ into O_2_. Researchers have developed various strategies to prepare nanozymes: 1) template methods: creating hollow or porous structures (e.g., using sacrificial templates) to increase the material’s surface area. 2) Solvothermal/hydrothermal synthesis: growing metal oxide nanocrystals under high temperature and pressure. 3) Pyrolysis of precursors: burning metal salts with organic ligands to form nanozymes. In addition, structural modulation techniques can significantly enhance catalytic performance, such as introducing oxygen vacancies (e.g., by doping Ti into CeO_2_) or surface functionalization with noble metals (e.g., loading Pt or Au onto carbonized metal-organic frameworks). Constructing Janus or layered multi-metal oxide structures can also endow nanozymes with multi-enzyme activities (Aldrich et al., 2023; Wang K et al., 2023).

Nanozymes exhibit very high catalytic efficiency and oxygen-generating capability. For example, Wang et al. reported Mn_3_O_4_ nanozymes with high catalase-like activity. These rapidly decomposed H_2_O_2_ and released large amounts of dissolved oxygen in a simulated OA joint environment (pH 6.5), effectively reducing intracellular ROS levels in chondrocytes (Wang W et al., 2023). In another study, researchers designed an ionically zwitterionic “hyaluronan synthase-mimicking” nanozyme (MPMP) that, under near-infrared excitation, generates a photothermal effect and releases Mg^2+^ ions. This design synergistically mimics SOD/CAT activity to scavenge ROS and release substantial O_2_, significantly promoting cartilage regeneration under inflammatory conditions (Yu et al., 2023). In another report, a polymer-coated Prussian Blue nanozyme (PPBzyme) was stable in physiological buffer and demonstrated long-term efficient oxygen release along with anti-inflammatory effects by inhibiting JNK phosphorylation (Cho et al., 2023). Overall, by finely tuning composition and structure, various nanozymes can achieve oxygen-release performance comparable to or even surpassing that of natural enzymes, providing new strategies for OA therapy.

### 3.2 Hydrogel-based oxygen-releasing materials

Given the joint’s fluidic environment, injectable hydrogel biomaterials have been extensively developed for OA therapy. Oxygen-generating hydrogels typically embed oxygen source particles or catalysts within a three-dimensional polymer network to achieve sustained oxygen release. Common oxygen sources include peroxides (such as CaO_2_ and MgO_2_), oxygen carriers (such as hydrogen peroxide or perchlorate salts), and nanozymes that catalyze H_2_O_2_ decomposition (Wang et al., 2022). Common methods to construct such hydrogels include: 1) physical embedding: directly mixing oxygen source microparticles into the gel precursor, so that upon gelation the particles are fixed within the network (Liu et al., 2022). 2) Enzymatic crosslinking: for example, using tyrosinase to crosslink carboxymethy chitosan while simultaneously incorporating CaO_2_ particles (Li et al., 2025). 3) Freeze-thaw gelation: using repeated freeze-thaw cycles of polymers like polyvinyl alcohol (PVA) to form a physically crosslinked network, trapping oxygen-releasing particles in its pores (Xu et al., 2025).

These oxygen-generating hydrogels, due to their polymer matrices, often exhibit prolonged oxygen release profiles. For example, Wang et al. constructed a responsive gel matrix encapsulating calcium peroxide (CaO_2_) in a degradable scaffold, which can release oxygen gradually according to the inflammatory environment. In simulated synovial fluid, this hydrogel continuously released oxygen for over a week, with peak dissolved oxygen levels reaching or exceeding those of normal tissues (Wang K et al., 2023). In another study, Zhang et al. developed an injectable hydrogel for OA using MXene-based nanoenzymes as the active core. In the presence of H_2_O_2_, this hydrogel continuously produces O_2_ while also providing anti-inflammatory effects (Zhang et al., 2024). Notably, recent studies often incorporate peroxides with stem cells, drugs, or growth factors within hydrogels, designing smart carriers that synergistically combine oxygen release with other therapeutic functions.

### 3.3 Microsphere-based oxygen-releasing materials

Oxygen-generating microspheres typically encapsulate unstable peroxides (such as CaO_2_ or MgO_2_) as a core to create a slow oxygen-release reservoir. These are commonly prepared through methods such as: emulsion–gelation, spray drying, and solvent evaporation, to produce oxygen-containing microspheres. To achieve stable and sustained oxygen release, microspheres are often fabricated with core-shell structures. For example, the microsphere surface may be coated with a dense polymer layer (such as PLGA) or an additional hydrophilic polymer shell to isolate the core from the surrounding water and control the peroxide reaction. By tuning the degradation rate and hydrophilicity of the shell, the core gradually hydrolyzes to generate H_2_O_2_, which is then slowly decomposed to release O_2_. For instance, one study encapsulated MgO nanoparticles within PLGA microspheres (MgO&SA@PLGA) and injected them into the joint cavities of OA rats. These significantly improved cartilage degeneration, even though the initial design was to release Mg^2+^, because the reaction of MgO with water also produced a small amount of oxygen, providing an additional oxygen supply (Zheng L et al., 2024). Similarly, another group loaded CaO_2_ into gelatin microspheres, which were then embedded in a self-assembling peptide and PLGA membrane composite scaffold. In this system, hydrolysis enabled continuous oxygen generation for approximately 21 days *in vitro* and 28 days *in vivo* (Zhang et al., 2021). Overall, oxygen-generating microspheres, through controlled chemical release 0.

### 3.4 Composite oxygen-releasing systems

Composite oxygen-release systems integrate oxygen sources with other functional components to achieve multi-modal therapy. In OA repair, oxygen sources are often combined with antioxidants (e.g., selenium compounds, plant-derived antioxidants), bioactive agents (e.g., stem cells, exosomes, anti-inflammatory drugs), or structural scaffolds. Assembly techniques include: 1) multilayer nano-composites: e.g., stacking layers of oxygen source particles, growth factors, and polymers. 2) Self-assembly: e.g., liposomes or nanospheres that co-encapsulate oxygen sources and drugs. 3) Covalent crosslinking: e.g., chemically attaching oxygen carriers to a polymer backbone.

These strategies can achieve concurrent oxygen release and other therapeutic functions. For example, co-loading an anti-inflammatory drug into an oxygen-releasing hydrogel can restore oxygen supply while simultaneously modulating inflammation. Alternatively, modifying the surface of oxygen-releasing microspheres with targeting ligands can enhance their retention and release efficiency within the joint. Chen et al. designed a biomimetic nanocapsule coated with an M2 macrophage membrane, carrying both uricase and a catalase-mimetic nanozyme. In this system, uricase degrades uric acid and the nanozyme catalytically converts the resulting H_2_O_2_ into O_2_. This dual-enzyme approach simultaneously provides oxygen supplementation and inflammation relief, demonstrating combined anti-inflammatory and oxygen-delivery effects (Chen et al., 2023). Similarly, some studies have also assembled CaO_2_ nanoparticles, antioxidant molecules (such as selenium nanoparticles) and scaffold materials to construct bone repair scaffolds with dual functions of oxygen release and oxidation resistance (García et al., 2024). The performance of such composite systems is evaluated not only by their O_2_ release profile but also by their antioxidant capacity (ROS scavenging efficiency), biocompatibility, and their ability to promote cell proliferation and differentiation. Overall, through multifunctional integrated design, composite oxygen-releasing systems achieve synergistic effects combining oxygen supply with drug therapy, anti-inflammation, or tissue support, providing new strategies for modulating the OA joint microenvironment and promoting tissue regeneration.

## 4 Oxygen supply, microenvironment modulation, and OA repair

Articular cartilage is an avascular tissue that exists in a chronically low-oxygen environment, disruption of its oxygen balance leads to increased oxidative stress, dysregulated HIF-1α signaling, and elevated inflammatory mediators, which accelerate cartilage degeneration (Lang et al., 2025). Lang et al. noted that by using strategies such as oxygen-generating nanomaterials to modulate joint oxygen levels, oxygen-related signaling pathways can be selectively activated to support cartilage repair (Lang et al., 2025). In line with this concept, recent studies have designed oxygen-releasing materials and confirmed that they can improve OA pathology through various molecular mechanisms. For example, Xiong et al. designed a pH-responsive degradable nanozyme (HMPBzyme) to mimic the OA microenvironment. This nanozyme suppresses the hypoxia-induced overexpression of HIF-1α and reduces intracellular ROS. *In vitro* and *in vivo*, HMPBzyme cooperatively protected mitochondrial function and downregulated HIF-1α, which shifted macrophages from a pro-inflammatory M1 phenotype toward an anti-inflammatory M2 phenotype, thereby remodeling the joint immune microenvironment. As a result, this nanozyme inhibited oxidative damage and hypoxia, significantly suppressing inflammation and promoting cartilage matrix synthesis, which improved cartilage degeneration in an OA rat model (Xiong et al., 2022). Zhao et al. developed an injectable oxygen-releasing hydrogel (L-MNS-CMDA) that continuously releases oxygen by decomposing endogenous or exogenous H_2_O_2_. This hydrogel promptly alleviated hypoxia-induced cellular oxidative stress and, by inhibiting macrophage M1 polarization, promoted M2 polarization and restored an anti-inflammatory immune microenvironment. In a knee cartilage defect model, implantation of this hydrogel modulated early inflammatory responses and significantly promoted the differentiation of bone marrow mesenchymal stem cells (BMSCs) into chondrocytes. This was evidenced by enhanced glycosaminoglycan and type II collagen expression, leading to effective cartilage tissue regeneration and functional recovery (Zhao et al., 2025). Zhou et al. reported a hyaluronic acid-based hydrogel microsphere (HAM-SA@HCQ) responsive to hypoxia and MMP-13, which rapidly degrades in OA lesions and releases the anti-inflammatory drug hydroxychloroquine (HCQ). The microsphere also contains ROS-scavenging structural components that synergistically eliminate free radicals and oxidative stress. Under hypoxic inflammatory conditions, degradation of the microspheres and release of HCQ significantly downregulated HIF-1α and various inflammatory factors in the joint and inhibited macrophage inflammatory activity. This system substantially lowered oxidative stress in the joint, prevented cartilage degradation, and thereby slowed OA progression (Zhou et al., 2024). Additionally, Wong et al. constructed a dual-crosslinked tyramine-alginate hydrogel containing CaO_2_ for sustained oxygen release. They found that the continuous generation of oxygen by CaO_2_ markedly maintained the viability of embedded cells and improved the gel’s mechanical strength and adhesiveness, thereby enhancing support for chondrocytes (Wong et al., 2022). This material promoted the chondrogenic differentiation of mesenchymal stem cells and the production of cartilage matrix, improving the efficiency of cartilage repair.

In summary, oxygen-releasing materials synergistically ameliorate the pathological state of OA through multiple mechanisms, including alleviating oxidative stress, modulating HIF-1α signaling, remodeling the immune-inflammatory microenvironment, and promoting chondrocyte differentiation and extracellular matrix synthesis.

## 5 Conclusion and perspectives

Oxygen-generating materials, by actively releasing oxygen and improving the local joint microenvironment, have shown significant promise for OA therapy. However, key limitations remain. First, limited oxygen release duration is a primary challenge. Most inorganic peroxides react rapidly under physiological conditions, making sustained release difficult, while excessively fast oxygen release can lead to hydrogen peroxide accumulation and hyperoxia toxicity, damaging cells (Montesdeoca et al., 2022). An ideal oxygen-generating system must precisely control the reaction rate within a safe range while providing oxygen for at least several weeks. Some studies have achieved over 2 weeks of continuous release by embedding oxygen sources in polymer microparticles or multilayer structures to slow hydrolysis (Jiang et al., 2024). For example, calcium peroxide-alginate microcapsules released oxygen continuously for 19 days in a rabbit model, significantly reducing local cell apoptosis. Nevertheless, further extending the release period and preventing burst release remain challenging (Wong et al., 2022). Second, local delivery efficiency and targeting are also critical issues. Materials injected into the joint cavity often disperse or are cleared rapidly, making long-term retention difficult. Therefore, developing carriers with controllable degradation and stimuli-responsiveness (such as hypoxia-triggered release) is necessary to ensure that oxygen is released locally at the lesion site rather than diffusing systemically. Additionally, safety evaluation is still inadequate: excessive oxygen or reactive oxygen species can inhibit angiogenesis or induce apoptosis. Thus, when designing oxygen-generating materials, combining them with antioxidants or enzymes (e.g., catalase) should be considered to balance oxygen release with peroxide removal (Zhao J et al., 2024).

Future research should focus on optimizing the preparation and functionality of oxygen-generating materials to better meet the needs of OA therapy. Directions include: 1) exploring novel oxygen sources and carriers: for example, using microbial photosynthesis, catalytic peroxide/H_2_O_2_ systems, or multifunctional nanocomposites to achieve controllable oxygen supply. 2) Developing smart responsive systems: materials that initiate oxygen release under hypoxic conditions or accelerate delivery during high oxygen demand could minimize wasted oxygen and side effects. 3) Investigating material-biology interfaces: evaluating how oxygen-generating materials affect immune cells (e.g., macrophage M1/M2 polarization) and studying the long-term biomechanical and biochemical feedback on chondrocytes and osteoblasts. 4) Strengthening *in vivo* and preclinical studies: establishing more complex OA animal models to evaluate joint structure repair, pain relief, and functional recovery, while conducting thorough biocompatibility assessments (histopathology, systemic toxicity, and immune responses). 5) Addressing scalability and formulation: developing cost-effective, scalable manufacturing methods and delivery systems for oxygen-generating materials to facilitate clinical translation and application (Jiang et al., 2024).

By addressing these challenges and research directions, the field can advance toward clinically viable oxygen-releasing therapies to improve the hypoxic microenvironment and repair mechanisms in osteoarthritic joints.
